# Does Subscapularis Repair Influence Range of Motion After Reverse Shoulder Arthroplasty With a Lateralized Glenosphere?

**DOI:** 10.7759/cureus.115445

**Published:** 2026-08-30

**Authors:** João Artur Bonadiman, Arthur Dutra Ricci, Carlos Rodriguez, Paulo Piluski, Osvandre Lech

**Affiliations:** 1 Orthopedics, Hospital São Vicente de Paulo/Instituto de Ortopedia e Traumatologia, Passo Fundo, BRA

**Keywords:** external rotation, internal rotation, lateralized glenosphere, range of motion, reverse shoulder arthroplasty, subscapularis repair

## Abstract

Background

The role of subscapularis repair in reverse shoulder arthroplasty remains controversial, particularly with contemporary glenoid designs that lateralize the center of rotation. This study compared early postoperative range of motion in patients undergoing reverse shoulder arthroplasty with and without subscapularis repair using a single prosthetic platform with a lateralized glenosphere.

Methods

This retrospective, comparative, single-center study included 66 patients who underwent primary reverse shoulder arthroplasty between January 2021 and December 2025 using the same prosthetic platform (Implantcast GmbH, Buxtehude, Germany) with a lateralized glenosphere and a 135° humeral neck-shaft angle. Forty-four patients underwent subscapularis repair and 22 did not. Active and passive elevation, external rotation, and internal rotation were assessed at three and six months postoperatively according to the standardized method of the American Shoulder and Elbow Surgeons. Between-group comparisons were performed for each range-of-motion variable at each time point, and paired observations were used to assess changes from three to six months. Twelve between-group comparisons were performed; the analysis was exploratory, no outcome was designated as primary, and Holm-adjusted p values are reported as a sensitivity analysis.

Results

At three months, passive external rotation was greater in the group without subscapularis repair (67.08 ± 12.15° vs. 57.65 ± 10.33°; difference −9.44°, 95% CI −18.03 to −0.85; p = 0.033). At six months, active internal rotation was greater in the repair group (median L1/T12 vs. L1; p = 0.023). Neither difference retained statistical significance after Holm-Bonferroni adjustment (adjusted p = 0.358 and 0.281, respectively). No other between-group difference was observed at either time point. Active elevation improved from three to six months in both groups (+19.5°, 95% CI +8.5 to +30.5 in the repair group; +29.1°, 95% CI +8.2 to +50.0 in the non-repair group), while within-group changes in internal rotation did not reach statistical significance in either group.

Conclusions

In this retrospective, non-randomized cohort, subscapularis repair was associated with nominal differences in early postoperative rotation after reverse shoulder arthroplasty with a lateralized glenosphere. Greater active internal rotation was observed at six months in the repair group, whereas greater passive external rotation was observed at three months in the non-repair group; however, neither finding remained statistically significant after adjustment for multiple comparisons. No overall range-of-motion advantage was identified for either strategy. These exploratory observations should be considered hypothesis-generating and require confirmation in adequately powered prospective studies.

## Introduction

Reverse shoulder arthroplasty has become a major surgical option for the treatment of several glenohumeral disorders, particularly rotator cuff tear arthropathy, irreparable rotator cuff tears associated with glenohumeral arthritis, complex proximal humeral fractures, and revision procedures [1,2]. Advances in prosthetic design and surgical technique have expanded its indications and have provided increasingly predictable outcomes, including pain relief, functional recovery, and high levels of patient satisfaction [3-5]. Despite these advances, several technical aspects remain controversial, including management of the subscapularis tendon during reverse shoulder arthroplasty [6-8].

The biomechanics of reverse shoulder arthroplasty are directly influenced by prosthetic design [9-11]. The original Grammont concept relied on medialization of the center of rotation to optimize deltoid function [2], whereas more contemporary designs have incorporated different strategies of lateralization [3,8,11]. Importantly, glenoid-side lateralization should be distinguished from humeral lateralization. Routman et al. classified a glenoid design as lateralized when the center of rotation is positioned more than 5 mm lateral to the glenoid face [11]. By increasing glenosphere thickness relative to its spherical radius, the center of rotation is shifted laterally from the glenoid face, which alters residual rotator cuff tension, deltoid wrapping, joint compression, impingement-free motion, and stability [11].

Within this biomechanical context, subscapularis repair remains one of the most debated issues in reverse shoulder arthroplasty. Proposed benefits include improved anterior stability, recovery of internal rotation, and preservation of muscle function, as reported by Friedman et al. [12], Franceschetti et al. [13], and Ameziane et al. [14]. Conversely, the biomechanical analyses of Berliner et al. [9] and the modelling study of Kontaxis and Johnson [10] suggest that repair may increase tension on the deltoid and posterior rotator cuff and may influence shoulder mechanics or limit selected motions. Clinical studies have reported conflicting findings: Clark et al. found no differences in pain, range of motion, or complications [15], while Roberson et al. concluded that repair was unnecessary with lateralized implants [16]. The systematic reviews and meta-analyses of De Fine et al. [17], Lachance et al. [18], and Bethell et al. [19] likewise found no consistent advantage, particularly when different prosthetic designs and heterogeneous patient populations were analysed together.

Although several studies have compared patients with and without subscapularis repair, many are characterized by methodological heterogeneity, including different prosthetic platforms, surgical techniques, multicenter populations, and rehabilitation protocols [17-19]. These differences limit direct comparison and make it difficult to isolate the effect of subscapularis repair on functional recovery after reverse shoulder arthroplasty. Studies evaluating this question within a single center, using the same prosthetic platform and similar perioperative protocols, remain relatively limited.

Therefore, the purpose of this observational, non-randomized, retrospective study was to compare active and passive range of motion at three and six months after reverse shoulder arthroplasty in patients treated with and without subscapularis repair using a single prosthetic platform with a lateralized glenosphere. These two assessment points correspond to the scheduled postoperative visits in our institutional protocol and bracket the transition to unrestricted strengthening at three months; they characterize early recovery and do not represent final outcome. No single range-of-motion variable was designated as a primary outcome; all six variables were analysed with equal weight at both time points, and the analysis is therefore exploratory.

We hypothesized that subscapularis repair would produce a directionally specific effect on early rotational recovery: that repair would be associated with greater recovery of active internal rotation, reflecting restoration of the principal internal rotator of the shoulder; that repair would be associated with a transient reduction in external rotation during the early postoperative period, reflecting restoration of anterior soft-tissue tension, with attenuation of this difference as the repair consolidated; that elevation, which depends principally on deltoid function, would not differ between groups; and that repair would not be associated with a higher rate of instability. Because the study is retrospective and no outcome was pre-specified, this hypothesis is stated as the mechanical expectation against which the observed pattern was interpreted, and not as a pre-registered confirmatory hypothesis; the analysis is accordingly exploratory throughout.

## Materials and methods

Study design and patient selection

This was an observational, retrospective, comparative, single-center study including consecutive patients who underwent primary reverse shoulder arthroplasty between January 2021 and December 2025. Inclusion criteria were: (i) primary reverse shoulder arthroplasty performed for primary glenohumeral osteoarthritis, rotator cuff tear arthropathy, fracture sequelae, or osteonecrosis; (ii) use of the same prosthetic platform with a lateralized glenosphere and a 135° humeral neck-shaft angle; (iii) surgery performed through a deltopectoral approach by a surgeon of the institutional Shoulder and Elbow Surgery Service; and (iv) at least one documented postoperative range-of-motion assessment at the three- or six-month visit. Exclusion criteria were: (i) revision reverse shoulder arthroplasty of a previous reverse implant; (ii) reverse arthroplasty performed with any other prosthetic platform or glenoid configuration; and (iii) absence of any documented postoperative range-of-motion assessment at either time point.

The three-month assessment was defined as the scheduled outpatient visit occurring at three months postoperatively within a window of ± 2 weeks, and the six-month assessment as the scheduled visit at six months within a window of ± 1 month. Assessments falling outside these windows were not used for the corresponding time point. The study was approved by the Research Ethics Committee of the Universidade Federal da Fronteira Sul (approval number 8.351.692; CAAE 85377024.2.0000.5564), and informed consent was obtained from all participants included in the study. Patients were identified through review of the institutional electronic medical record. All range-of-motion assessments were performed by the same evaluator, who was blinded to whether the subscapularis had been repaired.

Surgical technique and prosthetic design

All procedures were performed through a deltopectoral approach by surgeons from the same Shoulder and Elbow Surgery Service. All patients received the same reverse shoulder prosthetic platform manufactured by Implantcast GmbH (Buxtehude, Germany). The implant configuration combined a lateralized glenosphere with a 135° humeral neck-shaft angle. In this study, the term lateralized glenosphere refers specifically to glenoid-side lateralization. According to the classification proposed by Routman et al. [11], a center of rotation positioned more than 5 mm lateral to the glenoid face is considered a lateralized glenoid design. The glenosphere-based definition of lateralization was used to standardize the prosthetic design concept across the study population; the 135° humeral neck-shaft angle was considered an additional design characteristic of the construct rather than the criterion used to classify glenoid lateralization.

Subscapularis management

The subscapularis was managed using a peel technique when the tendon was present and could be mobilized. The feasibility of repair was assessed intraoperatively by the operating surgeon according to tendon presence, excursion, tissue quality, and the tension required to achieve repair. When these characteristics were considered favorable, the tendon was reinserted to the lesser tuberosity using three transosseous sutures with ULTRABRAID high-strength suture (Smith & Nephew, Inc., Andover, Massachusetts, USA), with the forearm maintained in a neutral position during fixation. Patients in whom the tendon was absent or could not be adequately mobilized or repaired without excessive tension were included in the nonrepair group. This standardized peel-and-transosseous repair technique was used for all patients in the repair group.

Rehabilitation protocol

The same institutional rehabilitation protocol was used for both groups. A sling with an abduction pillow maintaining 30° of shoulder abduction, with the forearm in neutral rotation, was used for three weeks in all patients irrespective of subscapularis management, according to the standard postoperative protocol of our service. This position avoids sustained adduction, internal rotation, and extension of the operated limb during the early postoperative period, relieves tension on the anterior soft tissues while a repair heals, and improves comfort during immobilization. Abduction-pillow immobilization is adopted by a substantial proportion of shoulder surgeons after reverse shoulder arthroplasty, although no consensus has been established regarding which type, position, or duration of postoperative immobilization is superior [20]. Immobilization was discontinued at three weeks so that supervised physical therapy could begin at four weeks postoperatively.

Beginning at four weeks postoperatively, physical therapy focused on progressive restoration of shoulder mobility and deltoid isometric exercises. Strengthening exercises were gradually introduced thereafter, with unrestricted strengthening initiated after three months postoperatively.

Range-of-motion assessment

Range of motion was assessed according to the standardized method adopted by the American Shoulder and Elbow Surgeons, which records the three cardinal planes of shoulder motion: elevation in the scapular plane, external rotation with the elbow near the side, and internal rotation referenced to spinal segments [21]. All assessments were performed with the patient standing.

Elevation was measured as maximal humerothoracic elevation in the scapular plane, with the goniometer fulcrum over the lateral aspect of the glenohumeral joint, the stationary arm aligned with the trunk in the mid-axillary line, and the moving arm aligned with the longitudinal axis of the humerus. External rotation was measured with the arm at the side and the elbow flexed to 90°, with the forearm in neutral rotation as the zero reference, the stationary arm of the goniometer directed anteriorly in the sagittal plane, and the moving arm aligned with the ulnar border of the forearm. Internal rotation was recorded with the patient reaching behind the back and was defined as the highest spinal segment reached by the tip of the thumb, identified by palpation of the spinous process; when the patient could not reach the spinal column, the level was recorded as the sacrum or the buttock. Active motion was assessed first, and passive motion was assessed subsequently by the examiner without patient effort. Angular measurements were assessed clinically and subsequently verified with a goniometer when applicable and were recorded in increments of 5°. Active and passive elevation, external rotation, and internal rotation were evaluated at three and six months postoperatively by the same evaluator, who was blinded to subscapularis management. Internal rotation results are presented as the median vertebral level with interquartile range (IQR); the statistical treatment of this variable is described below.

Not every range-of-motion measurement was available for every patient at every assessment, and the number of observations therefore varied according to the specific motion and follow-up time point. Two reasons accounted for the missing data. First, in this predominantly elderly population, a proportion of patients did not attend one of the two scheduled outpatient assessments within the corresponding visit window, and no measurement was recorded for that time point. Second, in some attended assessments, the clinical record documented active motion only, and passive motion was not consistently registered; this explains the smaller number of passive measurements available and the divergent trend between active and passive observations from three to six months. No measurement was omitted because the patient could not tolerate the examination, and pain was not a recorded reason for a missing measurement. Because the evaluator was blinded to subscapularis management, the availability of a measurement was independent of group allocation. No imputation was performed; all analyses used the observations available for each variable at each time point, and within-group comparisons were restricted to patients with paired measurements. At three months, measurements were available in 40 patients for active elevation, 32 for active external rotation, 29 for active internal rotation, 31 for passive elevation, and 29 for passive external and passive internal rotation. At six months, measurements were available in 46 patients for active elevation, 41 for active external rotation, 40 for active internal rotation, 18 for passive elevation and passive external rotation, and 17 for passive internal rotation.

Statistical analysis

Continuous range-of-motion variables (active and passive elevation and external rotation, measured in degrees) are reported as mean ± standard deviation. Between-group comparisons at each postoperative time point were performed with the independent-samples t-test, and within-group changes from three to six months were assessed with a paired t-test, including only patients with measurements available at both time points for the respective variable. Categorical variables are reported as counts and percentages and were compared with Fisher's exact test.

Internal rotation was recorded as the highest vertebral level reached and assigned ordinal ranks for statistical analysis, with higher ranks denoting greater internal rotation. Because the anatomic distance between consecutive vertebral levels is not uniform, internal rotation was treated as an ordinal variable and is reported as the median vertebral level with IQR, without any assumption of equal spacing between adjacent levels. Between-group comparisons of internal rotation were performed with the Mann-Whitney U test, and within-group changes from three to six months with the exact Wilcoxon signed-rank test, choosing the latter over the asymptotic approximation because of the small paired sample sizes and the presence of tied observations.

Throughout the tables, continuous variables measured in degrees are reported as mean ± standard deviation and ordinal internal rotation is reported as the median vertebral level with the interquartile range; the summary statistic applied to each variable is stated explicitly in a dedicated column of every table, and continuous and ordinal variables are presented in separate blocks.

No a priori sample size calculation was performed. All consecutive patients meeting the eligibility criteria during the study period were included, and the sample size was therefore determined by case availability rather than by a power calculation. Post hoc power was not computed, as observed power is a monotonic function of the observed p value and provides no additional information regarding the precision of the estimates; 95% confidence intervals are reported for all between-group differences in continuous variables instead.

Twelve between-group comparisons were performed (six range-of-motion variables at two time points), and twelve within-group comparisons of change from three to six months. No correction for multiplicity was applied to the primary analysis, as the study is exploratory and no outcome was designated as primary; adjustment for multiple testing is principally required in confirmatory analyses in which multiple test results must be combined into a single decision [22]. As a sensitivity analysis, the Holm-Bonferroni procedure was applied to the family of twelve between-group comparisons, and the adjusted p values are reported alongside the unadjusted values. The within-group comparisons were treated as a separate family, as they address recovery over time rather than a comparison between treatment strategies; for these, the magnitude of change with its 95% confidence interval is reported. All tests were two-tailed, and statistical significance was set at p < 0.05. Statistical analyses were performed using GNU PSPP, Version 1.4.1 (GNU Project, Free Software Foundation, 2021).

## Results

A total of 66 patients were included, of whom 44 (66.7%) underwent subscapularis repair and 22 (33.3%) did not. Mean age was 74.9 ± 5.9 years, and 55 patients (83.3%) were female. Demographic characteristics are presented by group in Table 1. The groups did not differ significantly in age (76.0 ± 6.1 versus 74.3 ± 5.8 years; t = −1.09, p = 0.281), sex distribution (p = 0.739), or operated side (p = 0.432). Surgical indications were not compared formally between groups, because a proportion of patients carried more than one diagnostic code in the electronic medical record and could not be assigned unambiguously to a single indication category; the heterogeneity of indication is addressed in the Limitations section.

**Table 1:** Demographic characteristics of the study population, by group Age is reported as mean ± standard deviation and compared with the independent-samples t-test; as in tables below, the t statistic is expressed as repair minus no repair. Sex and operated side are reported as counts and percentages of each group. For each of these two variables a single comparison of the overall distribution was performed with Fisher's exact test; the p value is therefore given once, on the variable heading row, and applies to both of its categories. Fisher's exact test does not yield a conventional test statistic, which is indicated by a dash. Surgical indications are not compared between groups because a proportion of patients carried more than one diagnostic code in the electronic medical record and could not be assigned unambiguously to a single indication category; this is addressed in the Limitations.

Variable	No repair (n = 22)	Repair (n = 44)	Total (n = 66)	Test statistic	p value
Age, years	76.0 ± 6.1	74.3 ± 5.8	74.9 ± 5.9	t = −1.09	0.281
Sex, n (%)	—	—		—	0.739
Female	19 (86.4%)	36 (81.8%)	55 (83.3%)	—	—
Male	3 (13.6%)	8 (18.2%)	11 (16.7%)	—	—
Operated side, n (%)	—	—	—	—	0.432
Right	15 (68.2%)	25 (56.8%)	40 (60.6%)	—	—
Left	7 (31.8%)	19 (43.2%)	26 (39.4%)	—	—

At three months postoperatively, the only between-group difference reaching nominal statistical significance was in passive external rotation, which was greater in the no-repair group (67.08 ± 12.15° vs. 57.65 ± 10.33°; difference −9.44°, 95% CI −18.03 to −0.85; t = −2.25, p = 0.033). No statistically significant differences were observed in active elevation, passive elevation, active external rotation, active internal rotation, or passive internal rotation (Table 2).

**Table 2:** Comparison of range of motion between groups at three months Internal rotation values are shown as the median vertebral level with the interquartile range in parentheses, presented in anatomical order from the more caudal (first quartile, indicating poorer internal rotation) to the more cranial level (third quartile, indicating better internal rotation). Levels were ranked so that a more cranial level denotes greater internal rotation (buttock < sacrum < L5 < L4 < L3 < L2 < L1 < T12 < T11 < T10 < T9 < T8 < T7). The values in parentheses are quartiles of the observed distribution and do not represent a predefined set of levels tested. When a median or interquartile limit fell between two adjacent vertebral levels, both levels are reported (e.g., L1/T12). The number of observations varies because not every range-of-motion measurement could be obtained in every patient at that follow-up assessment. Between-group comparisons were performed with the independent-samples t-test for continuous variables and the Mann-Whitney U test for internal rotation. Differences are expressed as repair minus no repair, with the 95% confidence interval of the difference; the t statistic is reported in the same direction, so that a positive value indicates a higher mean in the repair group. Confidence intervals are not reported for ordinal variables. Holm-adjusted p values were computed across the family of twelve between-group comparisons.

Variable	Statistic	No repair, n	No repair	Repair, n	Repair	Difference (95% CI)	Test statistic	p value	Holm-adjusted p
Active elevation (°)	Mean ± SD	12	117.50 ± 29.58	28	135.71 ± 34.90	+18.21 (−5.15 to +41.58)	t = +1.58	0.123	1.000
Active external rotation (°)	Mean ± SD	10	61.00 ± 9.66	22	61.14 ± 18.45	+0.14 (−12.57 to +12.84)	t = +0.02	0.983	1.000
Passive elevation (°)	Mean ± SD	12	143.33 ± 27.41	19	137.89 ± 24.40	−5.44 (−24.73 to +13.86)	t = −0.58	0.569	1.000
Passive external rotation (°)	Mean ± SD	12	67.08 ± 12.15	17	57.65 ± 10.33	−9.44 (−18.03 to −0.85)	t = −2.25	0.033	0.358
Active internal rotation (vertebral level)	Median (Q1–Q3)	9	L1 (L5 to L1)	20	L1 (L2/L1 to L1)	—	U = 67.5	0.254	1.000
Passive internal rotation (vertebral level)	Median (Q1–Q3)	12	L1 (L2/L1 to T12)	17	L1 (L2 to T12)	—	U = 98.5	0.892	1.000

At six months postoperatively, active internal rotation was greater in the subscapularis repair group (median L1/T12 vs. L1; U = 113.0, p = 0.023). No statistically significant differences were observed between groups for active or passive elevation, active or passive external rotation, or passive internal rotation (Table 3).

**Table 3:** Comparison of range of motion between groups at six months Internal rotation values are shown as the median vertebral level with the interquartile range in parentheses, presented in anatomical order from the more caudal (first quartile, indicating poorer internal rotation) to the more cranial level (third quartile, indicating better internal rotation). Levels were ranked so that a more cranial level denotes greater internal rotation (buttock < sacrum < L5 < L4 < L3 < L2 < L1 < T12 < T11 < T10 < T9 < T8 < T7). The values in parentheses are quartiles of the observed distribution and do not represent a predefined set of levels tested. When a median or interquartile limit fell between two adjacent vertebral levels, both levels are reported (e.g., L1/T12). The number of observations varies because not every range-of-motion measurement could be obtained in every patient at that follow-up assessment. Between-group comparisons were performed with the independent-samples t-test for continuous variables and the Mann-Whitney U test for internal rotation. Differences are expressed as repair minus no repair, with the 95% confidence interval of the difference; the t statistic is reported in the same direction, so that a positive value indicates a higher mean in the repair group. Confidence intervals are not reported for ordinal variables. Holm-adjusted p values were computed across the family of twelve between-group comparisons.

Variable	Statistic	No repair, n	No repair	Repair, n	Repair	Difference (95% CI)	Test statistic	p value	Holm-adjusted p
Active elevation (°)	Mean ± SD	19	142.63 ± 35.41	27	146.30 ± 33.87	+3.66 (−17.16 to +24.49)	t = +0.35	0.725	1.000
Active external rotation (°)	Mean ± SD	16	65.63 ± 15.37	25	64.60 ± 15.27	−1.03 (−10.94 to +8.89)	t = −0.21	0.835	1.000
Passive elevation (°)	Mean ± SD	5	132.00 ± 27.75	13	148.46 ± 31.58	+16.46 (−17.75 to +50.67)	t = +1.02	0.323	1.000
Passive external rotation (°)	Mean ± SD	5	60.00 ± 17.32	13	67.31 ± 20.06	+7.31 (−14.35 to +28.97)	t = +0.72	0.485	1.000
Active internal rotation (vertebral level)	Median (Q1–Q3)	16	L1 (L5 to L1)	24	L1/T12 (L1 to T8/T7)	—	U = 113.0	0.023	0.281
Passive internal rotation (vertebral level)	Median (Q1–Q3)	4	T11 (L1/T12 to T10)	13	T12 (T12 to T8)	—	U = 23.0	0.772	1.000

Both of the between-group differences reaching nominal significance - passive external rotation at three months and active internal rotation at six months - were identified among twelve between-group comparisons performed without correction for multiplicity. Following Holm-Bonferroni adjustment within this family, neither difference retained statistical significance (adjusted p = 0.358 and 0.281, respectively). These results are therefore reported as nominal, exploratory findings.

Because the median vertebral level does not convey the dispersion of individual results, the distribution of the highest vertebral level reached is presented by group and time point in Table 4. At six months, 12 of 24 patients in the repair group (50.0%) reached T12 or a more cranial level in active internal rotation, compared with 3 of 16 patients in the non-repair group (18.8%); 1 of 24 patients in the repair group (4.2%) and 3 of 16 in the non-repair group (18.8%) reached no level higher than the sacrum. These proportions are presented descriptively and were not subjected to additional significance testing.

**Table 4:** Distribution of the highest vertebral level reached in internal rotation, by group and time point Panel A presents active internal rotation and Panel B passive internal rotation. Values are the number of patients (percentage of patients with an available measurement in that group and time point) who reached each vertebral level as the highest level attained with the tip of the thumb behind the back. Levels are listed from the most cranial (greatest internal rotation) to the most caudal. Levels T9 and T11 were not recorded in any patient and are omitted. Denominators differ from the total group sizes because not every measurement was available at every assessment; the reasons are described in the Methods. These distributions are presented descriptively and were not subjected to significance testing.

Panel A — Active internal rotation				
Vertebral level	No repair, 3 months (n = 9)	Repair, 3 months (n = 20)	No repair, 6 months (n = 16)	Repair, 6 months (n = 24)
T7	0	4 (20.0%)	2 (12.5%)	6 (25.0%)
T8	0	0	0	1 (4.2%)
T10	0	0	1 (6.3%)	1 (4.2%)
T12	0	0	0	4 (16.7%)
L1	5 (55.6%)	11 (55.0%)	7 (43.8%)	10 (41.7%)
L3	1 (11.1%)	0	1 (6.3%)	1 (4.2%)
L4	0	1 (5.0%)	0	0
L5	2 (22.2%)	1 (5.0%)	2 (12.5%)	0
Sacrum	1 (11.1%)	0	1 (6.3%)	0
Buttock	0	3 (15.0%)	2 (12.5%)	1 (4.2%)

Panel B — Passive internal rotation				
Vertebral level	No repair, 3 months (n = 12)	Repair, 3 months (n = 17)	No repair, 6 months (n = 4)	Repair, 6 months (n = 13)
T7	1 (8.3%)	1 (5.9%)	0	3 (23.1%)
T8	0	1 (5.9%)	0	1 (7.7%)
T10	1 (8.3%)	1 (5.9%)	2 (50.0%)	2 (15.4%)
T12	3 (25.0%)	5 (29.4%)	1 (25.0%)	4 (30.8%)
L1	4 (33.3%)	4 (23.5%)	1 (25.0%)	2 (15.4%)
L2	1 (8.3%)	3 (17.6%)	0	0
L5	0	1 (5.9%)	0	1 (7.7%)
Sacrum	1 (8.3%)	0	0	0
Buttock	1 (8.3%)	1 (5.9%)	0	0

When the three- and six-month assessments were compared within each group, active elevation improved in both the repair group (+19.5°, 95% CI +8.5 to +30.5; Table 5) and the no-repair group (+29.1°, 95% CI +8.2 to +50.0; Table 6). Active and passive internal rotation showed a trend toward improvement from three to six months in the repair group that did not reach statistical significance (exact Wilcoxon signed-rank test: W = 45.0, p = 0.062 and W = 40.0, p = 0.062, respectively). No statistically significant changes were observed for the other evaluated variables.

**Table 5:** Within-group change from three to six months in patients with subscapularis repair Internal rotation is presented as the median vertebral level with interquartile range, in anatomical order from the more caudal to the more cranial level; when a value fell between two adjacent vertebral levels, both levels are reported (e.g., L1/T12). For internal rotation, change is reported as the median (interquartile range) of the paired differences in vertebral levels. The number of patients represents paired observations available at both three and six months for each variable. Within-group comparisons from three to six months were performed with the paired t-test for continuous variables and the exact Wilcoxon signed-rank test for internal rotation. Change and the paired t statistic are both expressed as the six-month minus the three-month value, so that a positive value indicates improvement. Within-group comparisons were treated as a separate family of hypotheses from the between-group comparisons and were not subjected to multiplicity adjustment; for active elevation, the magnitude of change is reported with its 95% confidence interval.

Variable	Statistic	n	3 months	6 months	Change (95% CI)	Test statistic	p value
Active elevation (°)	Mean ± SD	20	129.50 ± 35.91	149.00 ± 33.70	+19.50 (+8.50 to +30.50)	t = +3.71	<0.001
Active external rotation (°)	Mean ± SD	14	56.79 ± 19.47	64.29 ± 17.85	+7.50	t = +1.51	0.155
Passive elevation (°)	Mean ± SD	10	141.00 ± 26.85	147.00 ± 29.08	+6.00	t = +0.71	0.496
Passive external rotation (°)	Mean ± SD	10	60.50 ± 9.26	67.00 ± 20.58	+6.50	t = +0.96	0.364
Active internal rotation (vertebral level)	Median (Q1–Q3)	11	L1 (L5/L4 to L1)	L1 (L1 to T11)	0 (0 to 3.5)	W = 45.0	0.062
Passive internal rotation (vertebral level)	Median (Q1–Q3)	10	L1/T12 (L2/L1 to T12)	T11 (T12 to T9/T8)	0.5 (0 to 2)	W = 40.0	0.062

**Table 6:** Within-group change from three to six months in patients without subscapularis repair. Internal rotation is presented as the median vertebral level with interquartile range, in anatomical order from the more caudal to the more cranial level; when a value fell between two adjacent vertebral levels, both levels are reported (e.g., L1/T12). For internal rotation, change is reported as the median (interquartile range) of the paired differences in vertebral levels. The number of patients represents paired observations available at both three and six months for each variable. Within-group comparisons from three to six months were performed with the paired t-test for continuous variables and the exact Wilcoxon signed-rank test for internal rotation. Change and the paired t statistic are both expressed as the six-month minus the three-month value, so that a positive value indicates improvement. Within-group comparisons were treated as a separate family of hypotheses from the between-group comparisons and were not subjected to multiplicity adjustment; for active elevation, the magnitude of change is reported with its 95% confidence interval.

Variable	Statistic	n	3 months	6 months	Change (95% CI)	Test statistic	p value
Active elevation (°)	Mean ± SD	11	115.45 ± 30.12	144.55 ± 30.45	+29.09 (+8.18 to +50.00)	t = +3.10	0.011
Active external rotation (°)	Mean ± SD	9	60.00 ± 9.68	63.33 ± 16.39	+3.33	t = +0.56	0.591
Passive elevation (°)	Mean ± SD	4	147.50 ± 22.17	142.50 ± 17.08	−5.00	t = −0.32	0.769
Passive external rotation (°)	Mean ± SD	4	67.50 ± 5.00	60.00 ± 20.00	−7.50	t = −0.68	0.547
Active internal rotation (vertebral level)	Median (Q1–Q3)	8	L1 (L4/L3 to L1)	L1 (L4/L3 to L1/T12)	0 (−0.5 to 4)	W = 20.0	0.500
Passive internal rotation (vertebral level)	Median (Q1–Q3)	4	L1/T12 (L1 to T12/T11)	T11 (L1/T12 to T10)	0 (0 to 1.5)	W = 4.0	1.000

Postoperative complications and reinterventions observed during follow-up are summarized in Table 7. Two postoperative dislocations occurred: one in the nonrepair group and one in the repair group. The dislocation in the repair group occurred after a fall from standing height.

**Table 7:** Postoperative complications *One dislocation occurred in each group. The dislocation in the subscapularis repair group followed a fall from standing height. Frequencies are presented as absolute numbers and percentages of the total study population (n = 66).

Complication	Cases, n (%)
Acromial stress fracture	3 (4.5%)
Wound dehiscence without infection	1 (1.5%)
Postoperative dislocation	2 (3.0%)*

## Discussion

In this cohort, the two between-group differences reaching nominal statistical significance both involved rotational motion and occurred in opposite directions: greater passive external rotation in the non-repair group at three months and greater active internal rotation in the repair group at six months. Neither survived adjustment for multiplicity. Elevation recovered similarly in both groups. These observations are compatible with a reciprocal association between subscapularis management and the balance of internal and external rotation, but they were derived from multiple comparisons in a non-randomized retrospective cohort and are presented as preliminary and hypothesis-generating rather than as demonstrated effects.

The observed differences varied according to the movement analyzed. At six months, active internal rotation was greater in the repair group. External rotation showed an opposite pattern earlier in recovery, with greater passive external rotation in the no-repair group at three months. These differences were not reproduced across every assessment. In contrast, active and passive elevation followed a similar course in both groups, without clinically relevant between-group differences.

The predominance of differences in rotational motion is biomechanically plausible because the subscapularis is the principal internal rotator of the shoulder and contributes to dynamic glenohumeral balance [9,10]. Its repair may therefore favor recovery of internal rotation after reverse shoulder arthroplasty. Conversely, restoration of anterior soft-tissue tension may reduce external rotation excursion, particularly during the earlier stages of rehabilitation, which may help explain the greater passive external rotation observed at three months in patients without repair.

The time course of external rotation deserves specific comment. In the repair group, passive external rotation was lower than in the non-repair group at three months but increased thereafter (60.50 ± 9.26° to 67.00 ± 20.58°; p = 0.364), so that no between-group difference remained at six months. We interpret this as consistent with, rather than contrary to, the biomechanical expectation: the anterior restraint imposed by a healing subscapularis repair is greatest during the early rehabilitation period and attenuates as the repair consolidates and stretching progresses. The findings therefore suggest a transient rather than a permanent limitation of external rotation after repair. This interpretation should be considered exploratory, as the corresponding paired samples were small and the within-group changes did not reach statistical significance.

Conversely, the numerically lower passive external rotation observed in the non-repair group at six months compared with three months (67.08 ± 12.15° in 12 patients versus 60.00 ± 17.32° in 5 patients) should not be interpreted as a true loss of motion. These are largely non-overlapping measurements, and the corresponding paired analysis included only four patients (−7.50°; p = 0.547) with markedly increased dispersion at six months. A deterioration of passive external rotation after subscapularis release without repair would not be biomechanically expected, and we attribute this observation to the small number of paired passive measurements available at six months rather than to a clinical change.

The effect of repair is also unlikely to depend solely on whether the tendon is repaired. The conditions under which repair is achieved may be equally important, including tendon presence, excursion, tissue quality, and the tension required for fixation. In the present series, these factors were assessed intraoperatively and directly influenced the decision to perform repair. This individualized decision-making reflects clinical practice but also means that repair status may partly represent underlying differences in tendon quality and soft-tissue balance between groups.

This selective approach also has an interpretive advantage. Because repair was performed only when tendon presence, excursion, tissue quality, and repair tension were favorable, the repair group comprises shoulders in which a structurally sound and mechanically competent repair was achieved. Random allocation would necessarily have included tendons of poor quality repaired under tension, in which early failure would be expected, and would have diluted the effect under investigation. The greater active internal rotation observed at six months may therefore be more readily attributable to a functioning subscapularis than would have been possible under random assignment. This interpretive advantage does not, however, offset the corresponding loss of internal validity, and both readings of the same design feature are stated in the Limitations.

Interpretation of subscapularis repair should also account for the glenoid-side design of the reverse shoulder prosthesis. Routman et al. proposed a classification based on the position of the center of rotation relative to the glenoid face, defining a lateralized glenoid as a design in which the center of rotation lies more than 5 mm lateral to the glenoid face [11]. Glenoid lateralization increases residual rotator cuff tension and deltoid wrapping relative to medialized glenoid designs and may alter joint compression, stability, impingement, and rotational mechanics [11].

All patients in the present study received the same prosthetic configuration, combining a lateralized glenosphere with a 135° humeral neck-shaft angle. The designation of lateralization in this study is based on the glenoid side, consistent with Routman et al. [11], while the 135° humeral neck-shaft angle represents an additional feature of the implant construct that may also influence impingement-free motion and rotational biomechanics.

The present findings are consistent with part of the literature suggesting that subscapularis repair may be associated with improved recovery of internal rotation after reverse shoulder arthroplasty. Friedman et al. reported improved internal rotation and better functional scores in patients undergoing repair, without differences in complication or instability rates [12]. Franceschetti et al., in a study using a lateralized reverse shoulder platform, similarly reported favorable internal rotation after subscapularis repair [13]. More recently, Ameziane et al. also evaluated the effect of subscapularis repair on clinical outcomes after primary reverse shoulder arthroplasty [14]. Our observations follow this general pattern, although the difference we observed did not survive adjustment for multiple comparisons.

Other studies, however, have not demonstrated a consistent functional benefit from repair. Clark et al. found no relevant differences in pain, range of motion, or complication rates between patients with and without repair [15]. Roberson et al. concluded that subscapularis repair was unnecessary after reverse shoulder arthroplasty using lateralized implants [16]. The systematic review and meta-analysis by De Fine et al. found no clinically meaningful advantage associated with routine subscapularis repair, particularly in lateralized reverse shoulder designs [17].

In addition to the difference in internal rotation, the present study observed greater passive external rotation at three months in the no-repair group. Although this difference was no longer present at six months, it expands the discussion beyond internal rotation and raises the possibility that subscapularis management is associated with the overall rotational balance of the reconstructed shoulder. Differences in prosthetic design, repair technique, follow-up duration, and rehabilitation protocols likely contribute to the variability reported across studies [18,19].

A methodological strength of the present study is the use of a single center, a single shoulder surgery team, the same prosthetic platform, a lateralized glenosphere, a consistent 135° humeral neck-shaft angle, and a standardized rehabilitation protocol. These characteristics reduce several sources of variability frequently present in previous studies and may facilitate interpretation of the rotational differences observed between groups.

This study has several limitations. Its retrospective and non-randomized design limits control over data acquisition and introduces the possibility of selection bias and residual confounding. Although all range-of-motion assessments were performed by the same blinded evaluator, not every specific measurement could be obtained in every patient at each postoperative visit, resulting in variable sample sizes across outcomes and smaller paired samples for some three-to-six-month comparisons. This attrition was most pronounced for passive measurements at six months, which limits the interpretation of within-group changes in passive external and internal rotation.

The reliability of the range-of-motion measurements was not formally assessed. All measurements were obtained by a single evaluator who was blinded to subscapularis management, which removes inter-observer variability but leaves intra-observer reproducibility unquantified, as no repeated measurements were performed. Internal rotation carries a further intrinsic limitation: the highest spinal level reached behind the back reflects the combined contribution of glenohumeral internal rotation, scapulothoracic motion, elbow and wrist position, and trunk mobility, and cannot be attributed to glenohumeral rotation alone. The non-uniform anatomic spacing between adjacent vertebral levels was addressed by treating the variable as ordinal, but the composite nature of the measurement cannot be corrected statistically and should be borne in mind when interpreting differences expressed in vertebral levels.

Multiple between-group comparisons were performed without adjustment for multiplicity in the primary analysis, and the two statistically significant results emerged from twelve such comparisons. Neither retained significance after Holm-Bonferroni adjustment, and both should therefore be regarded as nominal findings. Although both involved rotational variables and followed the directions predicted by the mechanical function of the subscapularis, while elevation was unaffected, the possibility that they reflect chance cannot be excluded. These results are hypothesis-generating and require confirmation in an adequately powered prospective study.

A further limitation is the absence of patient-reported outcome measures. The present analysis was deliberately restricted to range of motion, and validated functional and satisfaction instruments were not collected; the difference in active internal rotation observed at six months could therefore not be correlated with patient-perceived function or satisfaction. This limitation is particularly relevant in the present population, in which 83.3% of patients were women with a mean age of 74.9 years, a group in which internal rotation governs activities such as perineal hygiene, fastening of garments, and reaching the mid-back. Whether a difference of one to two vertebral levels in active internal rotation is perceived as a meaningful functional gain therefore remains unanswered by our data, and the correlation between rotational recovery and patient-reported function is the specific objective of a prospective study currently being planned.

The study also included different clinical indications for reverse shoulder arthroplasty. Preoperative shoulder kinematics and functional patterns may differ among patients with degenerative disease, cuff-related pathology, and painful shoulder conditions [23,24], and these differences may influence postoperative recovery. The distribution of surgical indications between groups could not be compared formally, as a proportion of patients carried more than one diagnostic code in the electronic medical record and could not be assigned unambiguously to a single indication category. Residual imbalance in underlying pathology between the groups therefore cannot be excluded and constitutes a potential source of confounding by indication. The sample size and the ambiguity of the diagnostic coding did not permit a formal analysis of range of motion stratified by surgical indication, and such an analysis would have added substantially to the number of comparisons already performed. A comparison of recovery patterns across indications is a specific objective of the ongoing prospective follow-up of this cohort.

In addition, the decision to repair the subscapularis was based on intraoperative tendon characteristics rather than random allocation. Tendon presence, excursion, tissue quality, and repair tension may therefore have contributed to baseline differences between the groups and represent a potential source of selection bias or confounding by indication. This design therefore permits a more direct assessment of the effect of a competent repair, but the observed association should not be extrapolated to patients in whom repair would be achieved only under tension or in poor-quality tissue.

Finally, this was a single-center study using one prosthetic platform with a lateralized glenosphere and a 135° humeral neck-shaft angle. Although this consistency improves internal methodological homogeneity, the findings should be extrapolated cautiously to prosthetic configurations with different glenoid lateralization, humeral offset, or neck-shaft angles.

## Conclusions

In this retrospective, non-randomized cohort, subscapularis repair was associated with different early rotational findings after reverse shoulder arthroplasty with a lateralized glenosphere. Patients who underwent repair showed greater active internal rotation at six months, whereas patients without repair showed greater passive external rotation at three months. However, both findings were nominal and did not remain statistically significant after adjustment for multiple comparisons. No overall range-of-motion advantage was identified for either strategy, and active elevation improved within both groups during follow-up. These exploratory findings raise the possibility that subscapularis management may be associated with different patterns of postoperative rotational recovery, but causality cannot be inferred. Confirmation in adequately powered prospective studies incorporating patient-reported functional outcomes is warranted.
